# Dermoscopy of cutaneous metastasis of renal cell carcinoma

**DOI:** 10.1016/j.jdcr.2023.08.007

**Published:** 2023-08-19

**Authors:** Drew Kuraitis, Susan Pei

**Affiliations:** a Department of Dermatology, Tulane University, New Orleans, Louisiana; b Department of Dermatology, Roswell Park Comprehensive Cancer Center, Buffalo, New York

**Keywords:** angiogenesis, basal cell carcinoma, blood vessels, cutaneous metastasis, dermoscopy, metastasis, renal cell carcinoma, vasculature

## Clinical presentation

**Fig 1:**
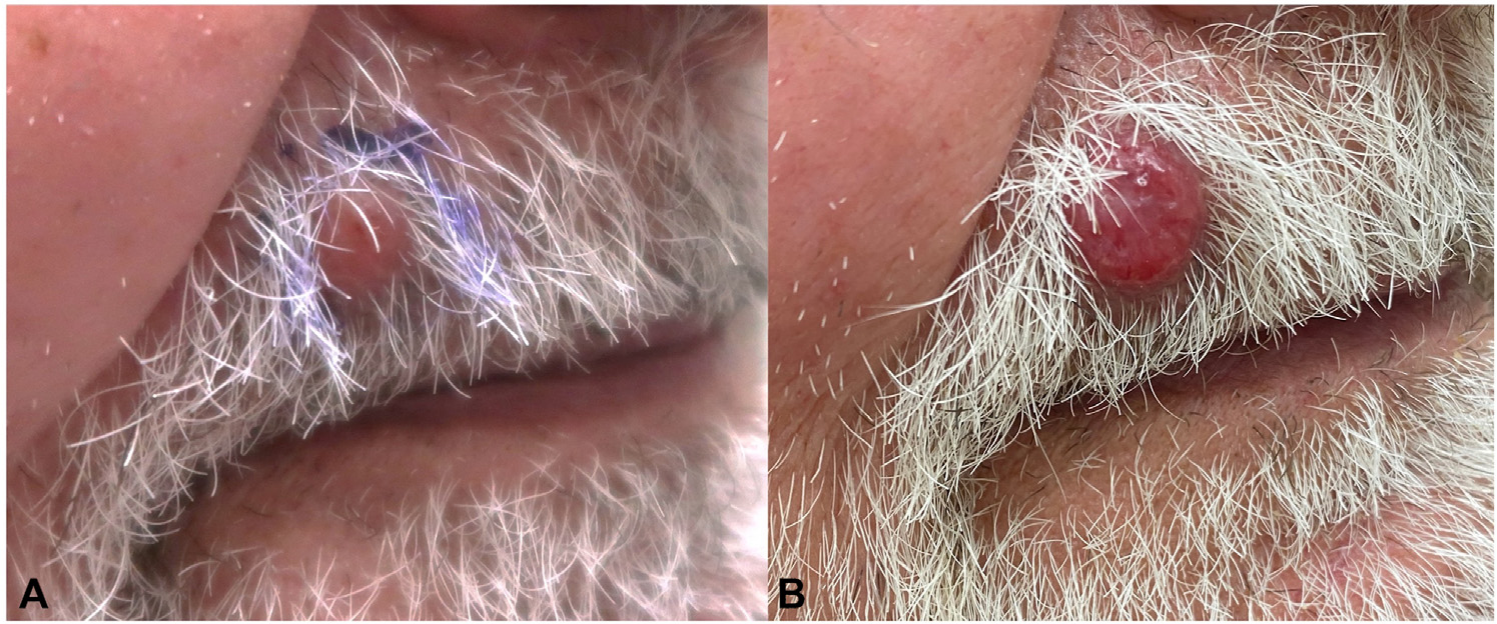
Initial appearance of renal cell carcinoma metastasis on the *right upper* cutaneous lip, flesh colored in appearance (**A**). Recurrent lesion 2 months after tangential biopsy, violaceous in color (**B**).

A 75-year-old man with a history of renal cell carcinoma (RCC) treated with nephrectomy 10 years prior presented with a 4-week history of an asymptomatic 5 mm nonviolaceous, translucent fleshy papule to the upper cutaneous lip (Fig 1, *A*). A tangential biopsy was consistent with RCC metastasis. Two months later, the patient returned with a recurrent lesion in the same location, although larger and violaceous in appearance (Fig 1, *B*).

## Dermoscopic appearance

**Fig 2:**
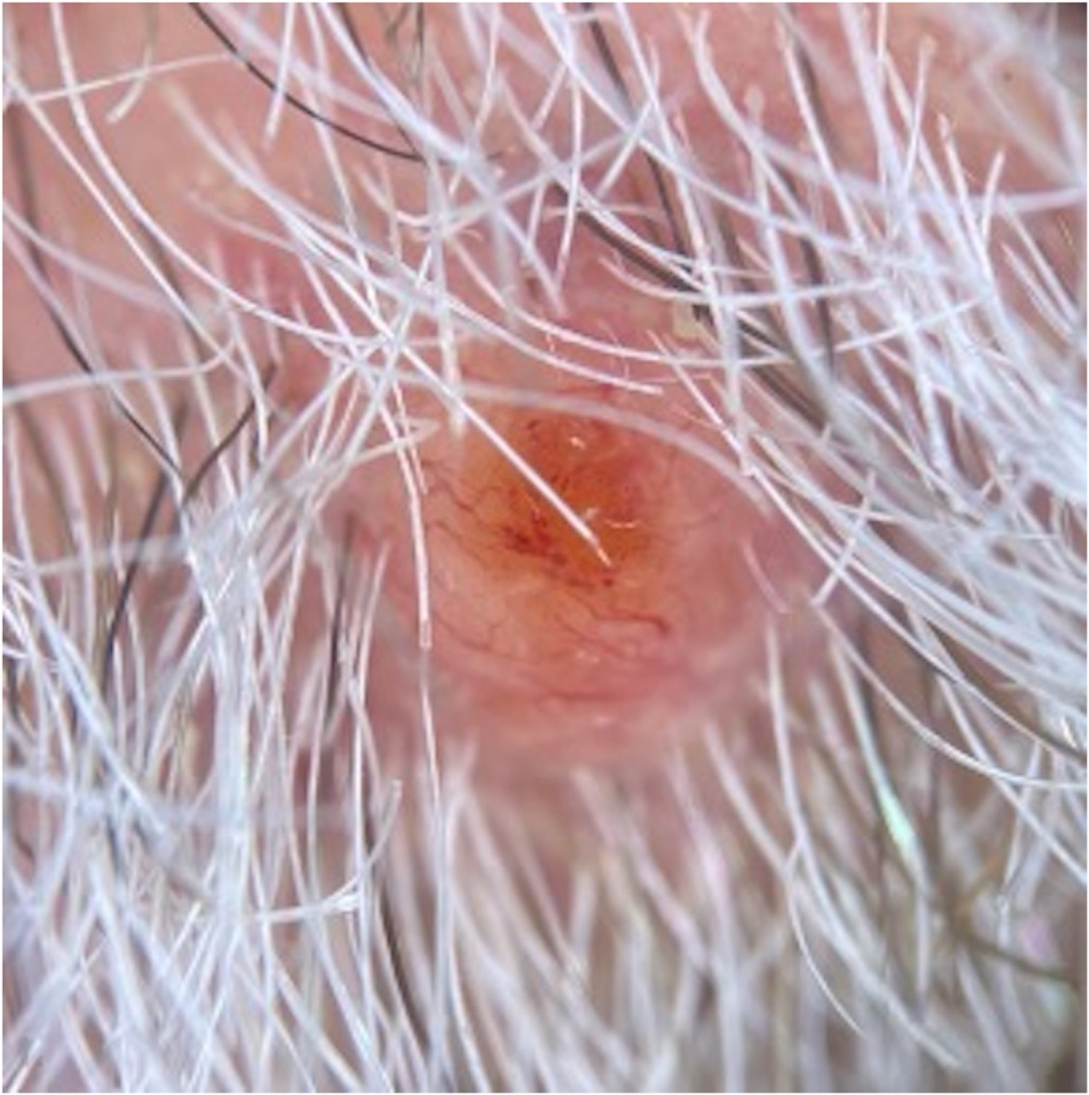
Dermoscopy of renal cell carcinoma metastasis at initial evaluation before biopsy. There are numerous arborizing and tortuous vessels throughout.

Dermoscopy initially revealed a translucent papule with tortuous and arborizing vessels throughout with centrally located lacunae (Fig 2).

## Histologic diagnosis

**Fig 3:**
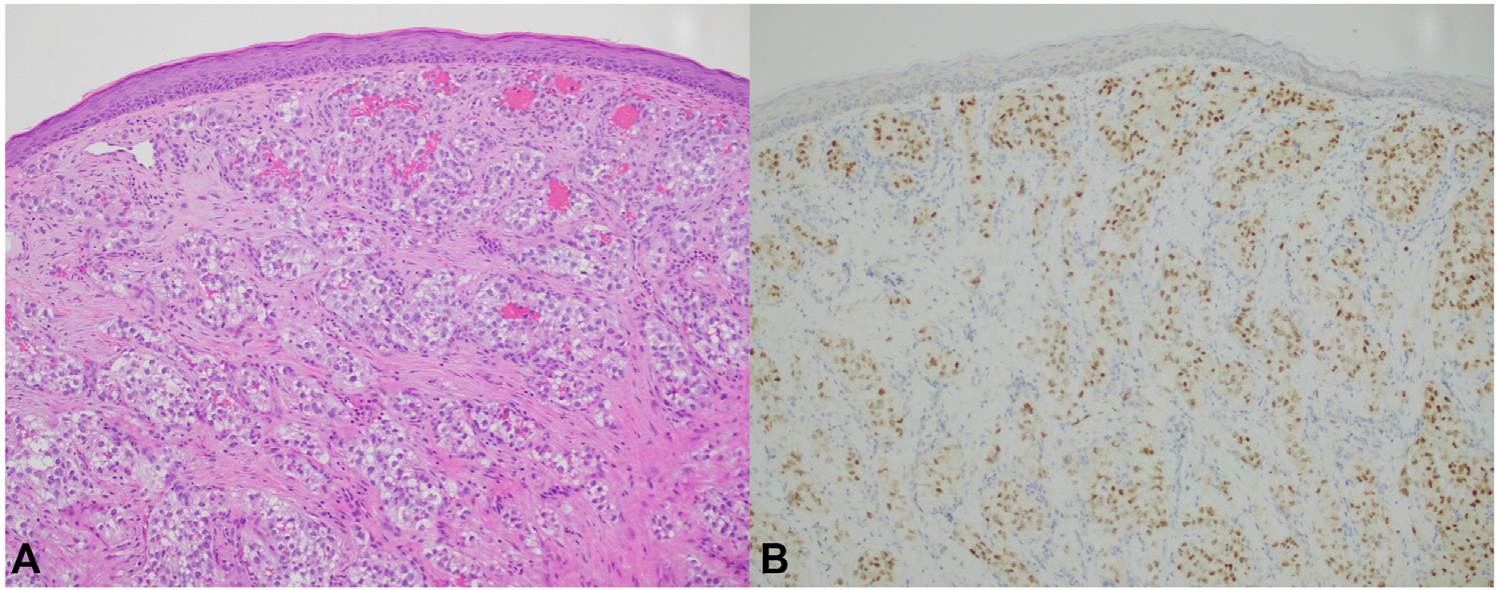
Tangential biopsy specimen of initial lesion. There are clear cells with hyperchromatic nuclei (**A**; hematoxylin and eosin, 100×) and diffuse nuclear positivity for PAX8 (**B**; 100×) within the dermis, characteristic of renal cell carcinoma.

Histopathologic examination demonstrated a dermal proliferation of atypical cells with clear cytoplasm (Fig 3, *A*) and diffuse nuclear positivity for PAX8 (Fig 3, *B*); a few ectatic superficial vessels are present, likely corresponding to vessels seen on dermoscopy.

**Key message**

Cutaneous metastases of RCC present as red-to-purple papulonodules favoring the head and neck.^1^ They may clinically mimic vascular neoplasms, melanoma, or basal cell carcinoma (BCC). The lesion presented here was unusual for RCC due to its initial fleshy color but subsequently regrew with a characteristic violaceous color of RCC metastases, likely due to extensive tumor vascularity. Arborizing vessels has a high positive predictive value for BCC and may be a characteristic feature of BCC.^2^ The lesion presented demonstrated arborizing and tortuous vessels, leading to inclusion of BCC on the differential diagnosis. Such a vessel arrangement should prompt biopsy to evaluate for malignancy, with an expanded differential to include BCC and RCC which may share similar dermoscopic findings.

## Conflicts of interest

None disclosed.
